# Microvascular status in juvenile Sjögren’s disease: the first nailfold videocapillaroscopy investigation

**DOI:** 10.1007/s10067-023-06857-5

**Published:** 2024-01-08

**Authors:** Adriano Lercara, Clara Malattia, Elvis Hysa, Marco Gattorno, Andrea Cere, Claudio Lavarello, Tamara Vojinovic, Emanuele Gotelli, Sabrina Paolino, Alberto Sulli, Carmen Pizzorni, Vanessa Smith, Maurizio Cutolo

**Affiliations:** 1 Laboratory of Experimental Rheumatology and Academic Division of Clinical Rheumatology, Department of Internal Medicine and Specialties (DIMI), University of Genova, IRCCS Ospedale Policlinico San Martino, Viale Benedetto XV, 6, 16132 Genova, Italy; 2 IRCCS Ospedale Policlinico San Martino, Genova, Italy; 3 Clinica Pediatrica E Reumatologia, IRCCS Istituto Giannina Gaslini, Genova, Italy; 4 Department of Neurosciences, Rehabilitation, Ophthalmology, Genetic and Maternal Infantile Sciences (DINOGMI), University of Genova, Genova, Italy; 5 Autoinflammatory Diseases and Immunodeficiencies Center, IRCCS Istituto Giannina Gaslini, Genova, Italy; 6 Department of Internal Medicine, Ghent University Hospital, University of Ghent, Ghent, Belgium; 7 Department of Rheumatology, Ghent University Hospital, University of Ghent, Ghent, Belgium; 8 Unit for Molecular Immunology and Inflammation, Flemish Institute for Biotechnology, Inflammation Research Center, Ghent, Belgium

**Keywords:** Connective tissue diseases, Juvenile Sjögren’s disease, Microcirculation, Microvascular damage, Nailfold videocapillaroscopy, Pediatric rheumatology

## Abstract

**Introduction:**

Juvenile Sjögren’s disease (jSjD) is a rare autoimmune disease characterized by exocrine gland involvement and systemic manifestations, including small vessel vasculitis and Raynaud’s phenomenon (RP). We aimed to investigate the microvascular status in jSjD patients by nailfold videocapillaroscopy (NVC) and the potential correlations with clinical and serological features.

**Methods:**

Clinical data from thirteen consecutive jSjD patients (11 females and 2 males), with a mean age of 16 ± 4 years, diagnosed before 16 years of age (mean age at diagnosis 12 ± 3) according to the 2016 American College of Rheumatology/EULAR criteria for adult SjD, were collected including age- and sex-matched healthy controls (HCs). Clinical, laboratory, and instrumental data were collected, together with NVC examination. Non-specific and specific NVC parameters were investigated, such as capillary density, capillary dilations, giant capillaries, microhaemorrhages and abnormal shapes. Associations between NVC findings and clinical/serological features were explored and analysed using parametrical and non-parametrical tests.

**Results:**

Capillary density reduction correlated significantly with articular involvement (arthralgias) (*p* = 0.024). Microhaemorrhages correlated with lower C3 levels (*p* = 0.034). No specific NVC pattern for jSjD was identified, whereas abnormal capillary shapes were significantly higher in jSjD patients than HCs (*p* = 0.005). NVC abnormalities were not associated with SjD-specific instrumental tests (biopsy, imaging, Schirmer’s test). RP was present in 8% of jSjD patients.

**Conclusions:**

The reduction of capillary density, as well as microhaemorrhages at NVC analysis, are significantly associated with some clinical aspects like articular involvement and serum biomarkers (C3 reduction). The NVC is suggested as safe and further analysis in jSjD patients.

**Supplementary Information:**

The online version contains supplementary material available at 10.1007/s10067-023-06857-5.

**Table:** 

**Key Points** *• Juvenile Sjögren’s disease (jSjD) exhibits a higher rate of abnormal shapes at nailfold videocapillaroscopy (NVC) compared to matched healthy controls.* *• Articular involvement in jSjD is significantly associated with a lower capillary number at NVC.* *• Microhaemorrhages on NVC are associated with lower C3 levels, prompting this finding as a putative novel negative biomarker/prognostic factor for jSjD.*		

## Introduction

Adult Sjögren’s disease (SjD) is a systemic autoimmune disease that causes the destruction of salivary, lacrimal and other exocrine glands, leading to xerostomia and xerophthalmia [1]. SjD is the second most frequent immune-mediated rheumatological condition in the USA [2, 3], but it remains rare in the paediatric age, with a prevalence ranging from 1 to 1.3% [3–5]. Both in the adult and in the paediatric population, there is a strong female predominance, with a frequency of 90% [2] and 80–87% [3, 5–7] respectively.

Childhood-onset or juvenile SjD (jSjD) is defined as a disease manifesting before 18 years of age [8, 9], with a peak age of 10–14 years [5]. No established and validated classification criteria are available for jSjD [9, 10], so that general practice is to use the American-European Consensus Group (AECG) criteria [11] and the American College of Rheumatology/European Alliance of Associations for Rheumatology (EULAR) criteria [12] for adult SjD. Child-specific criteria have been proposed [13], but they have not been validated and they have low sensitivity [14].

In jSjD, parotid swelling is very common [5, 9] and sicca symptoms like xerostomia and xerophthalmia often appear later in the course of the disease rather than at the onset [3, 9, 15, 16]. Extra-glandular manifestations include interstitial lung disease, renal disease (e.g. renal tubular acidosis), vasculitis, central nervous system (CNS) involvement, leukopenia, thrombocytopenia, anaemia, lymphadenopathy and musculoskeletal manifestations (arthralgia, arthritis, myalgia) [3, 5, 6, 8, 9]. Lymphoma is a rare complication, but still, it is reported in jSjD [17, 18].

Most children show positive antinuclear antibodies (ANA) and anti-SSA/Ro, and, to a lesser extent, anti-SSB/La [3, 6–8, 15] and rheumatoid factor (RF) are also frequently found [3, 5–7, 15], while low complement levels and cryoglobulinemia are less frequent [2, 3, 8].

Helpful tools in the diagnosis of jSjD are salivary gland biopsy, parotid ultrasound (US) and Schirmer’s test [5, 8, 9].

Although the histological hallmark of the disease is the focal lymphocytic sialadenitis [1], involvement of the microvascular system has been described, be it in the context of analysing the microvascularization of the salivary glands for potential new diagnostic strategies [19], or as a potential marker during the treatment with hydroxychloroquine [20].

Moreover, cutaneous vasculitis is one of the most common extra-glandular manifestation in adult SjD [21, 22]. Lastly, endothelial disfunction is seen [23, 24] and Raynaud’s phenomenon (RP) is occasionally present in adults in up to 13% [25], thus suggesting a further microvascular involvement [1, 5, 8, 9].

In this sense, nailfold videocapillaroscopy (NVC) represents a safe and non-invasive technique to investigate the status of peripheral microcirculation [26]. Indeed, some studies on NVC in adult SjD have been performed, reporting some alterations like reduced capillary density, dilated and giant capillaries, more frequently if RP was present [27–29], or even NVC scleroderma-pattern (“early” or “active”) in one-third of cases, although the NVC pattern does not apparently differ between those with positive and negative anti-SSa/Ro or anti-SSb/La antibodies or positive and negative salivary gland biopsy [30].

To our knowledge, no NVC studies on youngsters with jSjD have been performed, although efforts from the EULAR study group on microcirculation in rheumatic diseases have recently been made to standardize capillaroscopic findings in other autoimmune rheumatic diseases of children [31].

Therefore, the primary aim of this comparative cross-sectional study was to describe NVC findings in jSjD in order to investigate the presence of microvascular damage. Secondarily, clinical and serological correlations were searched in relation to NVC findings.

## Materials and methods

### Study population

Thirteen (13) consecutive jSjD patients referring to the Paediatric Rheumatology Unit of the IRCCS Istituto Giannina Gaslini of Genoa (Italy) who performed a NVC examination for their routine follow-up consultations were enrolled from June 2022 to January 2023.

jSjD patients were classified according to the 2016 American College of Rheumatology/EULAR criteria for adult SjD [12], with a diagnosis made before 16 years of age and were enrolled irrespective of sex.

Moreover, thirteen (13) consecutive healthy controls (HCs) both from the same Unit and from the Academic Division of Clinical Rheumatology of Genova University were enrolled.

### Clinical evaluation

Disease domains were dichotomously (yes/no) and retroactively assessed for all patients, and, in particular, sicca symptoms (xerostomia and xerophthalmia), parotid swelling and extra-glandular involvement, i.e. presence of RP, musculoskeletal disease (myalgia, arthralgia, arthritis), central nervous system involvement (cranial neuropathy, demyelination, stroke or transitory ischemic attack, convulsions, lymphocytic meningitis), peripheral nervous system involvement (axonal neuropathy, sensitive/motor neuropathy, vasa nervorum vasculitis), renal involvement (tubular acidosis, active sediment, renal failure, glomerulonephritis, renal vasculitis), lung involvement (persisting cough, interstitial disease, abnormal respiratory function tests), haematological cytopenias (leukopenia, autoimmune haemolytic anaemia, thrombocytopenia), lymphadenopathy.

Disease activity was established using the ESSDAI (EULAR Sjögren’s Syndrome Disease Activity Index) score [32].

Standard of care treatment for jSjD patients included oral glucocorticoids (prednisone) and conventional disease-modifying antirheumatic drugs (hydroxychloroquine, sirolimus).

### Laboratory tests

All jSjD patients underwent blood research for ANA and extractable nuclear antigen autoantibodies (ENA). ANA were evaluated by indirect immunofluorescence (IIFA) on Hep-2/liver cells (EUROPLUS ANA Mosaic FA 1510–1), with a 1:80 serum dilution as a cut-off value. Instead, ENA were tested through ELISA (EUROASSAY Anti-ENA ProfilePlus 1 ELISA IgG, EA 1590-1G). The kit ENA-Abs ELISA uses purified antigens of HEp-2 cell nuclei and nucleoli extract, spiked with highly purified antigens, Sm, RNP/Sm, SSa/Ro, SSb/La, Scl70 and Jo1. An enlarged panel was further prescribed to patients in case of a clinical suspicion of an overlapping scleroderma-spectrum disorder. In these subtypes of patients, antibodies against Scl-70, centromere A and centromere B, RNA polymerase III (RNAP-III), specifically RNAP-III 11 kDa (RP11) and RNAP-III 155 kDa (RP155), Ro-52, PM-Scl75, PM-Scl100, Ku, fibrillarin, Nor90, Th/To and Platelet-derived growth factor receptors (PDGF-R) were assessed.

Tests were performed according to the manufacturer’s instructions (EUROIMMUNAG, Lu beck, Germany). RF, complement C3 and C4 were also dosed.

Moreover, routine laboratory tests were performed, and these include full blood count (FBC), C-reactive protein (CRP) and erythrocyte sedimentation rate (ESR). Blood samples were collected, at most, 3 months before NVC examination.

### Instrumental examination

In order to perform the diagnosis of jSjD, one or more instrumental examinations were required. These included salivary gland biopsy (abnormal if evidence of focal lymphocytic sialadenitis with a focus score > 0 foci/4 mm^2^ — a focus is defined as a cluster of at least 50 lymphocytes [7]), Schirmer’s test (abnormal if tear production ≤ 5 mm/5 min) and parotid ultrasound (abnormal if hypoechogenity of parenchymal tissue or inhomogeneous glands were found [15]).

### NVC examination

NVC was performed in jSjD patients and HCs by the same physician blinded to the patient’s clinical history, using a 200 × magnification optical probe connected to an image analysis software (DS Medica Srl Videocap©, Ver 10.00.13, Milan, Italy) [33]. NVC was routinely performed to exclude microvascular alterations potentially associated to an overlap autoimmune disease.

As for standard protocol, each patient waited for a minimum of 15 min in a room at a temperature range of 20–22 °C before NVC. Two digital pictures of two-millimetre area in the middle of the nailfold bed of eight fingers, thumbs excluded, were collected for each subject [34].

According to the most recent data, the following capillaroscopic parameters were assessed: capillary number per linear mm (abnormal if capillary density < 7 capillaries/mm), capillary dilations (irregular or homogeneous increase of capillary diameter between 20 and 50 μm; capillaries with a diameter < 20 μm were defined as normal), giant capillaries (homogeneously dilated normal shaped loops with a diameter ≥ 50 μm), microhaemorrhages (due to hemosiderin deposit) and abnormal shapes (branched “bushy” capillaries, sign of neoangiogenesis, non-convex capillary tip, capillary crossing ≥ 3 times) [33, 34]. The validated semiquantitative rating scale by Cutolo et al. has been adopted to score each of the five NVC parameters mentioned (0, no changes; 1, < 33% of capillary alterations/reduction; 2, 33–66% of capillary alterations/reduction; 3, > 66% of capillary alterations/reduction per linear millimetre) [35].

In addition, the mean absolute capillaries count per linear millimetre (capillary density), was calculated with the same standardized methodology, considering all the 16 images collected for each subject [36].

The “scleroderma pattern”, if present, was assigned according to the 2019 Fast Track algorithm by Smith et al. [37].

### Ethics

This comparative cross-sectional study was conducted in accordance with the principles of the Declaration of Helsinki and Good Clinical Practice. At the time of consultations, all patients that had undergone NVC routinely as standard examination had been asked to provide a written informed consent for the realization of the exam and the utilization of their anonymized images and clinical data for research purposes. This study was conducted upon approval by the Ethical Committee by the University of Genoa and “Giannina Gaslini” Children Hospital, Genoa-Italy (no. 392REG2017).

### Statistical analysis

Continuous variables were reported as mean value and standard deviation (SD) or median and interquartile range (IQR) when appropriate, with categorical variables as count and percentage. Chi squared test or Kruskal–Wallis rank sum test was used to explore the heterogeneity of the characteristics by subject group. Fisher's exact test or Mann–Whitney test. Spearman’s rank correlation was used to calculate the relationship between ordinal variables, whereas Pearson’s correlation analysis was used for metrically scaled variables. Any *p* values equal or lower than 0.05 were considered statistically significant.

Datatab® Statistics Calculator was used for the statistical analysis.

## Results

### Patients’ demographics, clinical features and NVC parameters in jSjD

The whole cohort was composed by 26 patients, 13 jSjD patients and 13 healthy controls (HCs).

The demographic, clinical, laboratory, instrumental and treatment characteristics of jSjD patients are reported in Table 1.

**Table 1:** Descriptive analysis of the characteristics of the juvenile Sjögren’s disease patients’ cohort

Total patients, *n*	13
Sex, F (%)	11 (84%)
Age at diagnosis, years ± SD	12 ± 3
Age at NVC, years ± SD	16 ± 4
Disease duration, years ± SD	4 ± 5
ANA positivity, *n* (%)	10 (77%)
Anti-SSa/Ro positivity, *n* (%)	10 (77%)
Anti-SSb/La positivity, *n* (%)	9 (69%)
Rheumatoid factor positivity, *n* (%)	7 (54%)
CRP, mean ± SD, mg/l	0.3 ± 1.1
ESR, mean ± SD, mm/h	27 ± 19
C3, mean ± SD, mg/dl	120 ± 21
C4, mean ± SD, mg/dl	19 ± 3
Hb, mean ± SD, g/dl	12.6 ± 1.6
WBC, mean ± SD, *10^9^/L	5.0 ± 1.8
Neutrophils, mean ± SD, *10^9^/L	2.9 ± 1.6
Lymphocytes, mean ± SD, *10^9^/L	1.7 ± 0.4
PLT, mean ± SD, *10^9^/L	240 ± 50
Raynaud’s phenomenon, *n* (%)	1 (8%)
Parotid swelling, *n* (%)	8 (62%)
Xerostomia, *n* (%)	8 (62%)
Xerophthalmia, *n* (%)	7 (54%)
Arthralgia, *n* (%)	7 (54%)
Arthritis, *n* (%)	3 (23%)
Nervous system involvement, *n* (%)	1 (8%)
Lung involvement, *n* (%)	2 (15%)
Cytopenias, *n* (%)	4 (31%)
Vasculitis involvement, *n* (%)	1 (8%)
Muscular involvement, *n* (%)	1 (8%)
Adenopathy, *n* (%)	2 (15%)
Parotid US, abnormal (%)	10 (77%)
Schirmer’s test, abnormal (%)	7 (54%)
Salivary gland biopsy, abnormal (%)	9 (69%)
ESSDAI, mean ± SD	6 ± 5
Treatment with HCQ, *n* (%)	9 (69%)
Treatment with sirolimus, *n* (%)	3 (23%)
Treatment with PDN, *n* (%)	5 (38%)
Overlap with MTCD	2 (15%)
Overlap with SLE	1 (8%)

*F*, female; *SD*, standard deviation; *NVC*, nailfold videocapillaroscopy; *CRP*, C-reactive protein; *ESR*, erythrocyte sedimentation rate; *Hb*, haemoglobin; *WBC*, white blood cells; *PLT*, platelets; *ESSDAI*, EULAR Sjögren’s Syndrome Disease Activity Index; *US*, ultrasound; *HCQ*, hydroxychloroquine; *PDN*, prednisone; *MCTD*, mixed connective tissue disease; *SLE*, systemic lupus erythematosus

The mean disease duration was 4 ± 5 years with a mean onset age of 12 ± 3 years, while patients were meanly aged 16 ± 4 at the moment of NVC examination.

ANA positivity was reported in 10 patients (77%), all of them also positive for anti-SSa/Ro antibodies and 9 (69%) positive for anti-SSb/La antibodies. RF was found in 7 (54%) patients.

Parotid swelling was present in 62% of patients, as for xerostomia; xerophthalmia was present in 54% of patients. Musculoskeletal manifestations were of mild intensity and consisted of arthralgia (54%), mild arthritis (23%) and myalgia (8%).

Two patients had lung involvement (mild diffusing capacity for carbon monoxide reduction and restrictive pattern at spirometry), one had nervous involvement (peripheral paraesthesia and tremor) and another had vasculitis (manifested as purpura of the lower limbs). Only one patient had Raynaud’s phenomenon (8%).

Ten children (77%) had a parotid US compatible with SjD features, while salivary gland biopsy confirmed the diagnosis in nine patients. Schirmer’s test was abnormal in 54% of patients.

Patients were being treated with hydroxychloroquine (69%), sirolimus (23%) or prednisone (38%). Of note, no patient was under methotrexate, but seven of them (54%) were previously treated with it at disease onset because of articular involvement and interrupted because of side effects or primary inefficacy, with a mean time of treatment of 13 ± 8 months.

Moreover, 2 patients (15%) had overlap with mixed connective tissue disease (MTCD) and one (8%) patient with systemic lupus erythematosus, but SjD manifestations were the prevalent clinical picture.

**Fig. 1:**
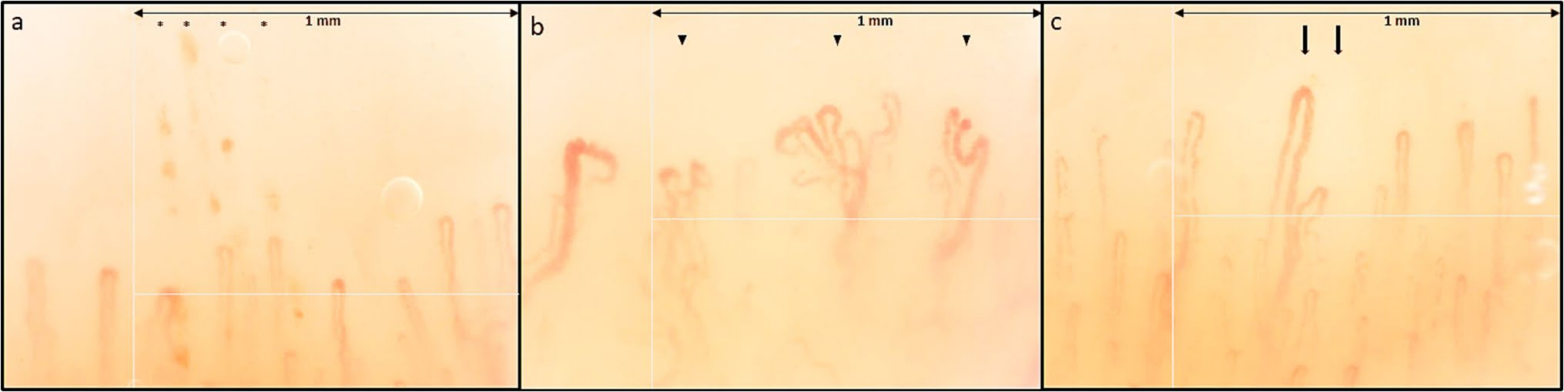
Examples of nailfold videocapillaroscopy images of three juvenile Sjögren’s disease patients (magnification 200 ×); (**a**) microhaemorrhages (*); (**b**) abnormal shapes (arrowheads) in a patient with overall reduced capillary density (6.3 capillaries/mm); (**c**) normal count of the capillaries of the first row, with two dilated capillaries (arrows). The patients presented non-specific alterations

Regarding the microvascular status, NVC was considered normal if only dilations < 33% of the total number of capillaries studied were present [35, 38]. Thus, 85% of NVC with non-specific abnormalities and no NVC with scleroderma pattern were observed. As for NVCs, data are reported in Table 2. Three images of non-specific NVC abnormalities in jSjD patients are depicted in Fig. 1.

**Table 2:** Demographic and capillaroscopic features of juvenile Sjögren’s disease (jSjD) patients and healthy controls (HC)

	jSjD	HC	*p*-value
Sex, F (%)	11 (84%)	8 (61%)	0.18
Age, mean ± SD	12 ± 3	13 ± 3	0.21
Capillary number, mean ± SD	8.5 ± 1	7.9 ± 0.8	0.12
Capillary number reduction, *n* (%)	1 (8%)	0 (0%)	1
Capillary dilations, *n* (%)	13 (100%)	13 (100%)	1
Giant capillaries, *n* (%)	0 (0%)	0 (0%)	1
Microhaemorrhages, *n* (%)	5 (38%)	7 (54%)	0.69
Abnormal shapes, *n* (%)	9 (69%)	2 (15%)	0.005*
Normal NVC, *n* (%)	2 (15%)	7 (54%)	0.1
Scleroderma pattern, *n* (%)	0 (0%)	0 (0%)	1
Non-specific abnormalities, *n* (%)	11 (85%)	6 (46%)	0.1

*F*, females; *SD*, standard deviation; statistically significant *p*-values < 0.05 are marked with * and reported in bold

No significant differences were detected between jSjD and HCs when categorised by age and sex, as shown in Table 2. In particular, 11 jSjD patients out of 13 (84%) and 8 HCs (61%) were female (*p* = 0.18); likewise, mean age for jSjD patients was 12 ± 3 and for HCs it was 13 ± 3 (*p* = 0.21).

Considering capillaroscopic findings in jSjD, the mean capillary count for jSjD was 8.5 (± 1) capillaries/mm. All patients presented with capillary dilations, 5 patients (38%) with microhaemorrhages and 9 (69%) with abnormal shapes. No giant capillary was reported in any group.

Therefore, jSjD patients showed statistically significantly more abnormal shapes than HCs (*p* = 0.005), while no differences were reported for the other NVC features (reduced capillary number, dilations, microhaemorrhages, giant capillaries).

### Associations between clinical features of jSjD patients and NVC findings

Since all jSjD patients presented with capillary dilations and none of them showed any giant capillaries, statistical analysis was not made and *p*-values were not calculated for these two features. Thus, only the mean capillary number, the presence of microhaemorrhages and the presence of abnormal shapes were considered for associations with clinical features.

As for age of the jSjD patients, no association was found with any of the NVC parameters analysed; the same can be said as for correlation with disease duration.

In the clinical domain, a statistically significant association was reported between the presence of arthralgia and the reduction of the mean capillary number (7.9 ± 0.9 vs 9.2 ± 0.8, *p* = 0.024).

Moreover, a statistically significant association was observed between a reduction of C3 levels and the presence of a higher number of microhaemorrhages on NVC (111.1 ± 19.6 in patients with microhaemorrhages vs 135.4 ± 13.6 in those without, *p* = 0.034). The other considered laboratory parameters (autoimmunity, inflammation, FBC) were not associated with NVC parameters.

No association was found between arthritis, parotid swelling, xerostomia, xerophthalmia, lung involvement, presence of lymphadenopathy and the ESSDAI.

Statistical analysis was not possible with RP, nervous system involvement, vasculitis involvement and muscle involvement as just one patient per group was present.

Finally, no instrumental test for jSjD (parotid US, Schirmer’s test and salivary gland biopsy) were found to correlate with NVC alterations.

Of note, no association was studied between NVC findings and treatments because this study lacks follow-up information, so we would not have been able to provide reliable statistics. These results are summarised in the Supplementary Table 1.

## Discussion

This is, to our knowledge, the first study which investigated the role of NVC in jSjD patients.

Interestingly, a significant association between the presence of articular involvement and a lower capillary density (*p* = 0.024), was observed, even though still in the range of the normal capillary number per linear millimetre.

The meaning of this finding is open to several possible explanations. Ideally, it would be useful and predictive if a patient with reduced capillary number were expected to develop articular involvement of the disease.

However, in our case, arthralgia or arthritis were present either as an anamnestic datum or at the moment of the NVC examination. The micro-traumatic origin of this finding cannot be excluded, either, since children often play intensively with their hands and the association of pain and NVC alterations can be the result of repeated hits or bumps.

On the other hand, this finding does not exclude the possibility to monitor capillary loss on NVC as a positive prognostic factor for the development of articular disease in jSjD, but further studies with larger cohorts are needed.

As a matter of fact, another study conferred a positive predictive role to the reduction of capillary density on NVC for the development of pulmonary disease in SjD [39].

We could not study deeply this association because only two patients presented with pulmonary disease and only one had pathologic (i.e., < 7 capillaries/mm) reduction of capillary density.

Although reduction of capillary density was reported in a single study on adult SjD patients [40], we only found one patient with capillary loss (mean 6.3 capillaries/mm) and our cohort presents a mean density of 8.5 ± 1 capillaries/mm (similar to the matched HCs).

Furthermore, the present finding of lower levels of C3 associated with higher frequency of microhaemorrhages in jSjD patients, may be in this sense worth investigating. On the other hand, the role of hypocomplementemia as a poor prognostic factor for SjD is well-recognized [1].

The presence of microhaemorrhages might be a potential poor prognostic imaging biomarker for jSjD, detected with a simple and non-invasive procedure. More studies are needed in order to confirm this hypothesis and subsequently to establish the cut-offs and/or distinctive morphology of the microhaemorrhages. A similar approach has been undertaken, for example, with anti-phospholipid syndrome, in which a typical comb-like disposition of the hemosiderin deposits has been noted at NVC [41]. In our paediatric cohort, only one patient exhibited comb-like microhaemorrhages, so it is not possible to date to think such NVC pattern as frequent in jSjD.

Indeed, these data may be interpreted as a possible signal of an overlapping syndrome with other CTDs and so NVC may have a role in monitoring patients at risk, like ANA and/or ACA positive patients or patients with RP [27, 33, 42]. This speculation derives from the data of a systematic literature review assessing NVC parameters in adult SjD [27].

As a further result, jSjD patients showed higher rates of capillaries with abnormal shapes compared to healthy controls, and this finding resulted statistically significant (*p* = 0.005). The term “abnormal shapes” is a standardized definition [34] that groups all the previous different ways to describe capillary morphological alterations (e.g. “ramifications”, “neo-angiogenesis”, “meandering”), in order to avoid confusing terminology.

A capillary with a typical “hairpin” shape, a (once or twice) crossing shape, or a tortuous shape is defined as “normal”, anything else is simply “abnormal” [33]. This dichotomic classification of the morphology of capillaries does not allow to reach an explanation to the altered morphology of capillaries seen on NVC, since a disease-specific “abnormal-shape pattern” remains elusive [33].

Non-specific abnormalities have been found [27–29], ranging our jSjD cohort in the non-scleroderma pattern NVC category, according to the 2019 “fast track algorithm” by Smith et al. [37].

The results confirmed that no specific NVC pattern for SjD can be described as it has been done in adults SjD patients [27–29], since no NVC-specific abnormalities (i.e. giant capillaries) or features compatible with a scleroderma pattern or scleroderma-like pattern were detected [43].

Even though about half of the adult SjD patients will only have one single vasculitic episode, vasculitis is however associated with more severe disease [21]. Furthermore, approximately 95% of vasculitis cases associated with SjD affect small vessels, specifically leukocytoclastic vasculitis [22]. This observation hints at the presence of a microcirculatory pathogenic route that is worth a thorough exploration by NVC.

RP, identified in 8% of young patients in our cohort, seems to mirror the adult SjD prevalence of 11–13% [25, 44]. These patients are also at increased risk of lung involvement and of positivity for anti-RNP and anti-centromere antibodies [25]. In this sense, a study on adult SjD has shown an association between anti-centromere antibodies positivity and a “scleroderma-like” pattern on NVC [42], but this can also indicate a possible overlap with other CTDs, such as systemic sclerosis or MCTD [33, 42].

To support the interest for the evaluation of the microvascular status in jSjD patients, the presence of isolated microhaemorrhages may reflect an indirect sign of endothelial distress and damage.

The microvascularization is increased, as a possible sign of inflammation, in salivary glands of adult SjD patients [19], whereas, in contrast, their retinal microvascular density is decreased [20].

Moreover, although rare, retinal vasculitis in adult SjD has been described and is mostly associated with anti-SSa/Ro or anti-SSb/La positivity [45]. Despite the fact that our cohort showed a higher prevalence of positive autoantibodies (77% ANA, 77% anti-SSa/Ro, 69% anti-SSb/La), limited sample size hindered correlation with any NVC features.

Notwithstanding the insights gained from our study, some limitations must be acknowledged. Our sample size, while suitable for preliminary retrospective exploration, restricted our ability to draw robust statistical correlations. Moreover, the variability among paediatric patients and the complex nature of SjD make it hard to point to exact cause-and-effect relationships with the NVC findings. Moving forward, a comprehensive approach involving larger prospective cohorts and multi-centre collaboration would be necessary in order to amplify the statistical power. Furthermore, our study design was cross-sectional, which limits our ability to infer causality between observed associations. Finally, a follow-up of these patients is needed in order to better understand the nature and meaning of the NVC findings.

The insights gathered from our investigation hold promising implications for both clinical practice and patient management. The identified association between abnormal capillary density, microhaemorrhages, capillary morphology, and specific disease manifestations provide valuable clues for risk assessment, phenotyping and prognosis also in jSjD (26).

While we could not definitively establish predictive markers in this study due to sample size constraints, the trends observed are worth further investigation.

In real life, specialists in SjD may use these findings to tailor therapeutic strategies and improve disease monitoring.

## Conclusions

This comparative cross-sectional study has revealed new insights into the microvascular aspects of jSjD patients evaluated at NVC never evaluated before.

The statistically significant link between the reduction of capillary density and joint involvement requires further exploration.

The association between lower C3 levels and higher frequency of microhaemorrhages at NVC analysis introduces to a new path for a putative additional prognostic factor.

Future investigations involving larger-scale studies and a standardized NVC approach may provide validation for these preliminary results.

### Supplementary Information

Below is the link to the electronic supplementary material.

Supplementary file1 (DOCX 22 KB)

## Data Availability

The datasets generated and/or analysed during the current study are not publicly available for ethical and privacy reasons but they are available from the corresponding author on reasonable request.
