# Supplementary material for: Microvascular status in juvenile Sjögren’s disease: the first nailfold videocapillaroscopy investigation
Source: Clin Rheumatol. 2024 Jan 8;43(2):733–41. doi: 10.1007/s10067-023-06857-5 (PMC10834566; doi:10.1007/s10067-023-06857-5)
Supplement: Supplementary file 1 — Supplementary file1 (DOCX 22 KB) [file 10067_2023_6857_MOESM1_ESM.docx]

**Supplementary Table 1.** Association analysis between juvenile Sjögren’s disease patients’ features and specific NVC parameters. NA=not applicable; NVC=nailfold videocapillaroscopy; CRP=C-reactive protein; ESR=erythrocyte sedimentation rate; Hb=haemoglobin; WBC=white blood cells; PLT=platelets; ESSDAI=EULAR Sjögren’s Syndrome Disease Activity Index; US=ultrasound; statistically significant p-values < 0.05 are marked with ***** and are reported in **bold**.

|  | Microhaemorrhages | | | | Abnormal shapes | | | | | Mean capillary number | | |
| --- | --- | --- | --- | --- | --- | --- | --- | --- | --- | --- | --- | --- |
|  | present | absent | | p-values | present | | absent | | p-values | r value or means ± SD | | p-values |
| Age | 15±2.6 | 17±4 | 0.383 | | 15.3±3.7 | 18.2±3.9 | | 0.440 | | r= 0.13 | | 0.673 |
| Disease duration | 2.8±4.2 | 4.5±4.9 | 0.534 | | 3.2±4.6 | 5.3±4.6 | | 0.660 | | r= -0.02 | | 0.945 |
| ANA positivity | 4/10 | 6/10 | 0.900 | | 6/10 | 4/10 | | 0.210 | | 8.5±1.2 vs 8.6±0.6 | | 0.906 |
| Anti-SSa/Ro positivity | 4/10 | 6/10 | 0.937 | | 6/10 | 4/10 | | 0.217 | | 8.5±1.2 vs 8.6±0.6 | | 0.906 |
| Anti-SSb/La positivity | 3/9 | 6/9 | 0.710 | | 6/9 | 3/9 | | 0.710 | | 8.4±1.2 vs 8.7±0.5 | | 0.704 |
| Rheumatoid factor positivity | 2/7 | 5/7 | 0.534 | | 4/7 | 3/7 | | 0.181 | | 8.8±1 vs 8.2±1 | | 0.330 |
| CRP | 3±1.2 | 3.6±2.3 | 0.453 | | 3.3±2 | 3.7±1.7 | | 0.732 | | r= -0.19 | | 0.534 |
| ESR | 22±11.8 | 30±22.7 | 0.472 | | 30.7±21.3 | 19.11.2 | | 0.320 | | r= -0.3 | | 0.929 |
| C3 | 111.1±19.6 | 135.4±13.6 | **0.034*** | | 117.7±14.4 | 126.8±33.4 | | 0.690 | | r= -0.14 | | 0.658 |
| C4 | 19.4±2 | 18.4±3.8 | 0.596 | | 18.3±2 | 19.8±5.3 | | 0.783 | | r= 0.09 | | 0.763 |
| Hb | 12.2±1.3 | 12.7±1.8 | 0.580 | | 12±1.6 | 13.7±1.3 | | 0.274 | | r= 0.08 | | 0.790 |
| WBC | 4.5±1 | 5.4±2.1 | 0.445 | | 5.3±2.1 | 4.5±0.9 | | 0.733 | | r= -0.15 | | 0.623 |
| Neutrophils | 2.4±0.9 | 3.1±1.9 | 0.622 | | 3±1.9 | 2.5±0.6 | | 0.814 | | r= -0.19 | | 0.541 |
| Lymphocytes | 1.7±0.2 | 1.7±.0.5 | 0.982 | | 1.7±0.4 | 1.5±0.3 | | 0.627 | | r= -0.06 | | 0.836 |
| PLT | 258.8±49.2 | 228.1±50.2 | 0.303 | | 233.7±40 | 254±73.7 | | 0.547 | | r= 0.46 | | 0.116 |
| Raynaud’s phenomenon | 0/1 | 1/1 | 0.615 | | 1/1 | 0/1 | | 0.923 | | NA | | NA |
| Parotid swelling | 2/8 | 6/8 | 0.354 | | 6/8 | 2/8 | | 0.435 | | 8.3±1.1 vs 8.9±0.8 | | 0.290 |
| Xerostomia | 3/5 | 2/5 | 0.943 | | 5/9 | 4/9 | | 0.534 | | 8.5±0.8 vs 8.5±1.4 | | 0.918 |
| Xerophthalmia | 2/7 | 5/7 | 0.534 | | 4/7 | 3/7 | | 0.622 | | 8.6±1 vs 8.4±1.1 | | 0.725 |
| Arthralgia | 3/7 | 4/7 | 0.836 | | 6/7 | 1/7 | | 0.138 | | **7.9**±0.9 vs 9.2±0.8 | | **0.024*** |
| Arthritis | 0/1 | 1/1 | 0.371 | | 1/2 | 1/2 | | 0.217 | | 8.3±0.6 vs 8.7±1.1 | 0.219 | |
| Nervous system involvement | 0/1 | 1/1 | 0.615 | | 0/1 | 1/1 | | 0.308 | | NA | NA | |
| Lung involvement | 1/2 | 1/2 | 0.769 | | 2/2 | 0/2 | | 0.769 | | NA | NA | |
| Cytopenias | 1/4 | 3/4 | 0.600 | | 2/4 | 2/4 | | 0.330 | | 8.2±0.1 vs 8.6±1.2 | 0.290 | |
| Vasculitis involvement | 0/1 | 1/1 | 0.615 | | 0/1 | 1/1 | | 0.308 | | NA | NA | |
| Muscular involvement | 0/1 | 1/1 | 0.615 | | 1/1 | 0/1 | | 0.923 | | NA | NA | |
| Adenopathy | 1/5 | 4/5 | 0.715 | | 2/9 | 7/9 | | 0.305 | | 9.1±0.1 vs 8.4±1.1 | 0.358 | |
| Parotid US | 3/10 | 7/10 | 0.371 | | 8/10 | 2/10 | | 0.217 | | 8.5±1.2 vs 8.4±0.5 | 0.859 | |
| Schirmer’s test | 3/7 | 4/7 | 0.836 | | 3/7 | 4/7 | | 0.138 | | 8.7±1 vs 8.2±1.1 | 0.446 | |
| Salivary gland biopsy | 3/9 | 6/9 | 0.710 | | 6/9 | 3/9 | | 0.800 | | 8.4±1.1 vs 8.6±0.9 | 0.600 | |
| ESSDAI | 4.4±4.8 | 7.6±5.7 | 0.316 | | 7.1±4.9 | 4.8±6.9 | | 0.611 | | r= 0.06 | 0.847 | |
